# A DNA-Based Plasmonic Nano-Ruler

**DOI:** 10.3390/ijms26062557

**Published:** 2025-03-12

**Authors:** Aura Cencini, Mary Bortoluzzi, Graziano Rilievo, Federica Tonolo, Fabio Vianello, Massimiliano Magro, Alessandro Cecconello

**Affiliations:** Department of Comparative Biomedicine and Food Science, University of Padua, Viale dell’Università 16, 35020 Legnaro, PD, Italy; aura.cencini@phd.unipd.it (A.C.); mary.bortoluzzi@phd.unipd.it (M.B.); graziano.rilievo@unipd.it (G.R.); federica.tonolo@unipd.it (F.T.); fabio.vianello@unipd.it (F.V.); alessandro.cecconello@unipd.it (A.C.)

**Keywords:** DNA nanotechnologies, self-assembly, plasmonic nanoparticles, fluorescence, quenching–enhancing

## Abstract

DNA is an exceptional building block for the fabrication of dynamic supramolecular systems with switchable geometries. Here, a self-assembled, tunable plasmonic–fluorescent nanostructure was developed. A precise sliding motion mechanism was operated through the control of strand displacement reactions, shifting two single-strand DNA (ssDNA) rails connected by a ssDNA quasi-ring structure. The system was reconfigured as a nano-mechanical structure, generating six discrete configurations, and setting specific distances between a tethered gold nanoparticle (AuNP) and a fluorophore, Sulfo-Cyanine3 (Cy3). Each configuration produced a distinct fluorescence emission intensity via plasmonic quenching/enhancement effects, and therefore the structure behaved as a nano-ruler. To optimize the system, the reversible distance-dependent fluorescence quenching or enhancement phenomena were investigated by testing AuNPs with diameters of 5, 10, and 15 nm, yielding the best performances with 10 nm AuNPs. Furthermore, a geometric model of the system was produced, confirming the observed results. The fluorophore–plasmonic surface positioning, conferred by the DNA ruler, led to a finite state nano-machine with six alternative signal outputs. This mechanism, working as a fluorescent reporter, could find application in a multiple-responsive detection system of single-strand nucleic acids, such as viruses or microRNAs.

## 1. Introduction

DNA has been extensively used to tailor functional supramolecular systems using Watson–Crick simple base-pairing rules [1]. In the boundless scenario of DNA technology, machine-like dynamic assemblies, that can be structurally switched in a programmable way, stand out for their potential of exerting a punctual control over matter through the generation of structures and mechanical forces at an actual nano-size level [2,3,4]. In this view, a kaleidoscope of artificial DNA structure-based applications is available in the literature nowadays. Among them, DNA-based nanoscale templating of inorganic objects [5], multi-responsive DNA hydrogels [6], and other DNA-based nano-machines [7,8,9] were produced through self-assembly or mechanically “fuelled” designs. This consists of using ssDNA not only as a structural material but also as a “fuel”, namely. Auxiliary single strands of DNA are added to the solution to induce motion through conformational changes and, as a molecular motor, each cycle produces duplex DNA as a waste product [10,11,12]. Such DNA-based nano-mechanical structures, that can be reversibly opened and closed in a programmed way, were applied to develop finite state machines and logic gates [13], paving the way for DNA computing [14] and storage systems [15,16,17].

Fluorescence-based nanotechnologies, that commonly found application in a wide range of scenarios, ranging from biosensing to in vivo imaging [18,19,20,21], were successfully used as reporting systems for the reconfigurations of DNA-based machines [22]. In this context, aiming at improving the limit of detection and to expand the choice of fluorophores, continuous efforts were dedicated to the enhancement of fluorescence intensity [23]. Indeed, among different approaches, molecular fluorescence could be modulated through the vicinity of a metal nano-surface, resulting in emission quenching at short distances or emission enhancement farther away from the surface and at a precise distance range [24,25,26]. Theoretical analyses demonstrated that several parameters including metal type, fluorophore dipole orientation, and nanoparticle shape are key players, besides surface–fluorophore distance, for manipulating fluorescence [27]. In recent years, DNA origami nano-rulers for super-resolution fluorescence microscopy have evolved from experimental proof-of-concepts to commercially available benchmarks [28]. These self-assembled nanostructures are dynamic supramolecular systems that are obtainable with high reproducibility and high yields, arranging a defined number of fluorophore molecules within programmed nano-sized architectures, commonly referred to as the “scaffold”. Furthermore, the requirement of a molecular frame for the precise positioning of nanoparticles and fluorophores was explored by the tailored assembly of DNA-scaffolded metal nanoparticle/fluorophore nanostructures in one- [29], two- [30], or three-dimensional structures [31].

Here, we explore a novel DNA actuator combining (i) the ability of DNA as a building block to develop dynamic nano-machines and (ii) the effect of noble metal nanoparticles on a fluorophore optical properties. The DNA structure behaves as a six-state dynamic spacer comprising a DNA-functionalized single fluorophore (Cy3) and a single DNA-modified AuNP, tethered to sterically dictated positions via sequence-specific DNA duplex formation. The functionalized parts are designed to “slide” over each other, shortening or extending the fluorophore–AuNP separating distance by strand displacement mechanism-triggered reconfigurations, therefore affecting fluorescence emission. For these reasons, the structure is also called “slider”, here on.

We demonstrate that 10 nm AuNPs are the ideal plasmonic counterpart for the fine-tuning of the Cy3 fluorescence. The reversibility of the fluorescence quenching/enhancement phenomena upon DNA-based motion mechanisms is supported by fluorescence emission levels during the strand displacement-triggered scaffold reconfigurations. Six discrete fluorophore–AuNP distances and as many specific fluorescence signals highlight the feasibility of the complex assembly as a finite state fluorescent nano-device, providing information on the fluorophore–AuNP separating distance via fluorescence emission and thus behaving as a nano-ruler.

## 2. Results

The positioning of nano-objects at close relative distances relies on the ability to precisely control their chemical connectivity. DNA allows for a dictated control over the formation of sequence-specific duplexes, which in this study was used to determine the location of a fluorophore with sub-nanometer precision. On the other hand, to ensure the attachment of individual AuNPs on the scaffold and avoid crosstalk with other DNA components or multiple bindings, single DNA-functionalized particles are necessary, i.e., monovalent particles. This was obtained via thiol-gold chemistry using an -SH-modified oligonucleotide (see Tables S1 and S2, Supplementary Materials, for a list of the DNA strands), and by electrophoretic separation and purification of the monovalent product. To optimize the system as a fluorescence reporter, the effects of 5, 10, and 15 nm diameter AuNPs on a fluorophore (Cy3) optical properties were investigated. For simplicity, the results section is dedicated to the description of the best configuration of the system, with 10 nm AuNPs. A brief comparison of the 10 nm AuNP effects with the 5 and 15 nm AuNP-DNA hybrid assemblies is commented at the end of the section (see also Supplementary Materials, Figure S1).

**Figure 1:**
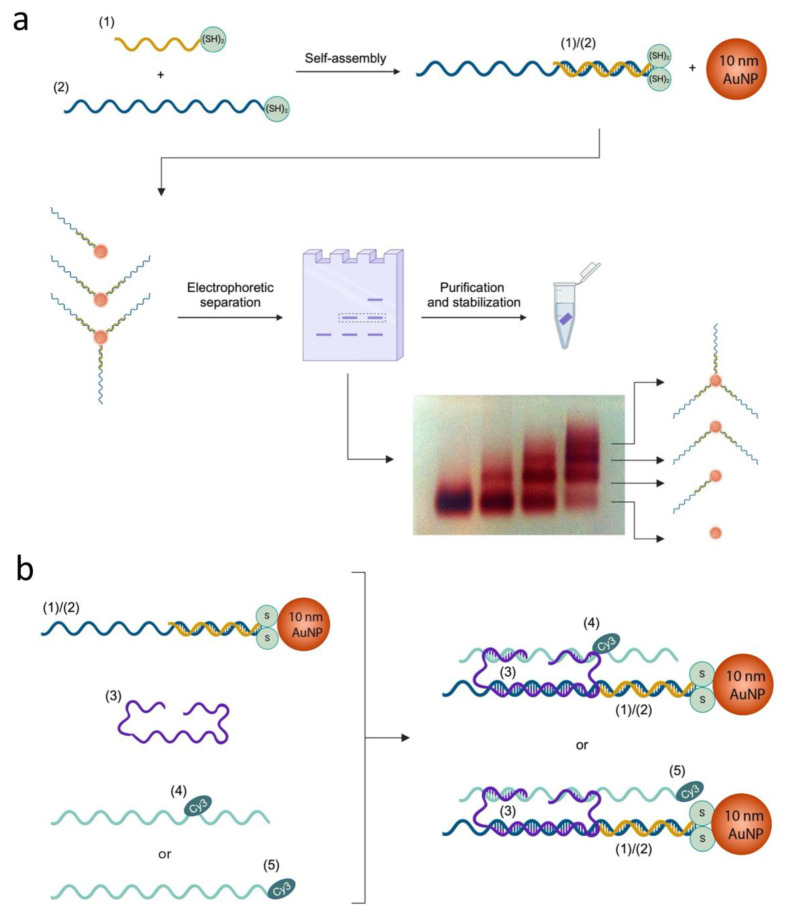
DNA slider assembly scheme. (**a**) Production of single DNA-functionalized 10 nm AuNPs and representative agarose gel electrophoretic separation, showing samples at different (1)/(2): AuNP ratios; at high ratios (lanes on the right side), several bands are observed containing AuNPs carrying zero, one, two, or three (1)/(2) duplexes (arrows point at the respective structure). (**b**) Stepwise assembly scheme of slider DNA scaffold; Rail B was either strand (4) or (5), with Cy3 as an internal or 3′ modification, respectively. DNA strands (1), (2), (3), (4), and (5) are represented with different colors for clarity. For the respective sequences, see Supplementary Materials, Tables S1 and S2.

Figure 1a shows a scheme of the thiolated-DNA functionalization process and an exemplary gel electrophoretic separation of the DNA-functionalized 10 nm AuNPs, where bands corresponding to one-, two-, and three-strand functionalized particles are visible at the increasing thiolated-DNA/AuNP molar ratio. Once the band of interest was excised from the agarose gel, particles were collected in a dialysis tube and cleaned in TBE buffer (a complete description of the AuNP modification, separation, and purification is reported in the Materials and Methods Section, while additional details are reported in the Supplementary Materials).

The self-assembled dynamic DNA structure generating the nano-ruler comprises AuNP-modified Rail A, i.e., partial duplex (1)/(2), the roller ssDNA quasi-ring strand (3), and Rail B, i.e., Cy3-functionalized ssDNA (4) or (5). The structure exists in six configurations: States S1, S2, and S3, carrying a Cy3 functionality internal to strand (4). Strand (5) has the same sequence as strand (4) but with Cy3 positioned at the 3′ end, realizing states S4, S5, and S6. The dynamic reconfigurations are operated by additional strands (6), (7), and (8) and their respective complementary strands (6*), (7*), and (8*) via the toehold-assisted strand displacement mechanism. Strands (9) and (10) rigidify the structure, limiting the oscillations and allowing for precise fluorophore–plasmonic surface relative positioning. Figure 1b shows a stepwise scheme of the DNA structure self-assembly. A typical self-assembly process of the nano-ruler DNA scaffold is associated with a 50–80% yield, as already reported [8], while AuNP decoration was assumed to produce the final product with a high yield (close to 100%) due to the excess of DNA-modified AuNPs used (see also Supplementary Materials). It should be noted that the final structure was not purified, and small amounts of defective assemblies are expected to be present in the final solution.

Once the slider was assembled, its reconfigurations were monitored in a quartz cuvette by fluorescence emission changes (λ = 565 nm) upon the addition of a small excess of the appropriate strand (for the details of the reconfiguration operations, including concentrations and volumes of all mixtures, see the methods section and the Supplementary Materials). It should be noted that the emission wavelength was selected as the maximum emission of the fluorophore. In addition, Cy3 is considered a resilient emitter in terms of photobleaching, which makes it the elective choice for long experiments.

**Figure 2:**
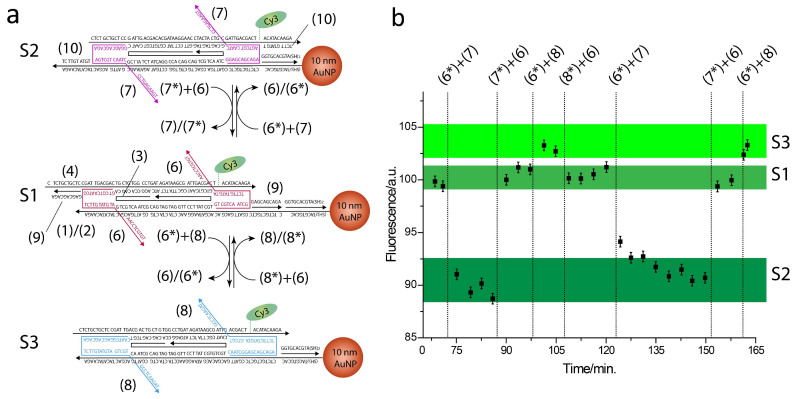
Mechanical control of the DNA slider fluorescence properties through strand displacement reactions. (**a**) Three discrete configurations S1, S2, and S3 were obtained, generating three specific Cy3-AuNP separating distances. Operating DNA strands (6), (7), and (8) are colored red, pink, and blue, respectively, and the complementary sequences are indicated as (6*), (7*), and (8*). (**b**) Fluorescence emission levels recorded at (λ = 565 nm) associated with slider reconfigured across states S1, S2, S1, S3, S1, S2, S1, and S3.

A scheme of the slider reconfigurations across states S1, S2, and S3 is reported in Figure 2a along with the respective fluorescence emission changes, in Figure 2b. At first, the slider was locked in state S1 by strand (6) and rigidifying helper strand (9) (see Table S2, Supplementary Materials, for a list of the DNA sequences of the operating and rigidifying oligonucleotides). In this state, Cy3 is the farthest from the AuNP surface and near-field phenomena are highly inefficient. We set this emission intensity as the normalizing value, as reported in Figure 2b plot. Upon the addition of complementary strand (6*) and strand (7), strand (6) is removed by the formation of the more stable duplex (6)/(6*), while strand (7) locks the slider in state S2. Here, the fluorophore is the closest to the AuNP surface and the respective fluorescence signal reaches a minimum. Next, state S1 is restored by adding removal strand (7*) and strand (6). In the following step, when strands (6*) and (8) are added to the mixture, the slider reconfigures to state S3, bringing Cy3 at an intermediate distance from the AuNP, and the associated fluorescence emission reaches a maximum. Finally, state S1 is restored by adding removal strand (8*) and strand (6). The slider was cycled one more time across states S2, S1, and S3, showing a near perfect reproducibility of the first cycle emission levels (Figure 2b).

**Figure 3:**
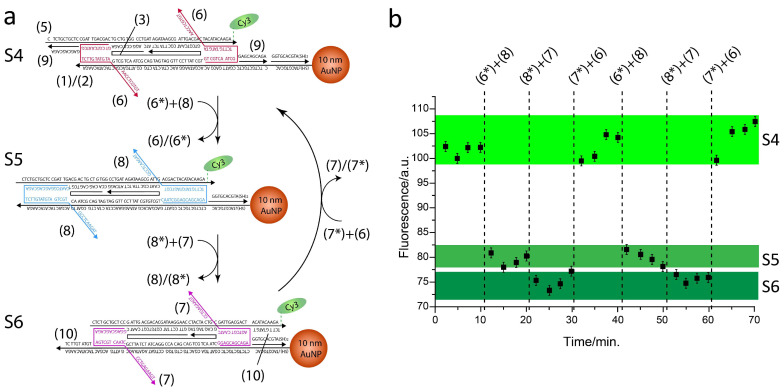
Mechanical control of fluorescence properties of the nano-ruler through strand displacement reactions. (**a**) Three discrete configurations S4, S5, and S6 were obtained, generating three specific Cy3-AuNP separating distances. Operating DNA strands (6), (7), and (8) are colored red, pink, and blue, respectively, and the complementary sequences are indicated as (6*), (7*), and (8*). (**b**) Fluorescence emission levels recorded at (λ = 565 nm) associated with slider reconfigurations across states S4, S5, S6, S4, S5, S6, and S4.

Next, a slider where Rail B strand (4) was replaced with strand (5) was assembled and characterized, similarly to the first slider. Here, Cy3 is positioned at the 3′ end of strand (5) and therefore the slider reconfigurations are expected to generate structures with shorter Cy3-AuNP separating distances. Figure 3a shows a scheme of the slider reconfigurations across states S4, S5, and S6 along with the associated fluorescence emission levels (Figure 3b). The slider was initially locked in state S4 by strand (6) and the stepwise addition of strands (6*) + (8) and (8*) + (7) reconfigured the slider to states S5 and S6, respectively. Fluorescence emission levels were progressively reduced, indicating quenching phenomena as Cy3-AuNP separating distance became shorter. In addition, as previously observed, the slider was re-set to state S4 and cycled again through states S5, S6, and S4, showing results analogous to the first cycle.

For comparison, 5 and 15 nm AuNP nano-rulers were assembled, and the characteristic fluorescence emission levels were recorded at the different structure states. The 5 nm AuNPs were ruled out as an efficient plasmonic material as they gave rise to a negligible enhancement effect, likely ascribable to their small size. On the other hand, the larger 15 nm AuNPs were not suitable for this molecular configuration as indicated by their imprecise quenching/enhancement responses (Figure S1, Supplementary Materials). This can be attributed to the nanoparticle diameter, associated with broader oscillations in the current system, making fine-positioning impossible in the range of distances set by the DNA slider. These outcomes suggest that an adequately dimensioned design is required for matching with the steric hindrance of 15 nm, probably needing a bulkier DNA scaffold that would prevent particle oscillations. Hence, 10 nm AuNPs emerged as the optimal metal counterpart for fabricating a fluorophore–plasmon surface distance-depending finite state nanomachine, potentially applicable as a multi-responsive fluorescent reporter for in vitro tests.

## 3. Discussion

Fluorescence imaging and sensing have greatly benefited from the integration of surface plasmon resonance, yielding a number of surface plasmon-assisted fluorescence tools with noteworthy applications such as, among others, the real-time visualization of biomolecules in vivo [32].

Here, we developed a real-time reporter of single-strand nucleic acid, such as virus or microRNA, using a fine-tunable plasmonic–fluorescent nano-ruler. Indeed, single-strand nucleic acid targets can be detected through strand displacement reactions with complementary sequences in the ruler, inducing the precise sliding motion of two ssDNA rails over an ssDNA quasi-ring structure. This mechanism operates a fine-tuning of the distance separating a 10 nm AuNP and a fluorophore unit (Cy3), with an effect on Cy3 fluorescence that ranges from quenching to enhancement, generating six discrete signal outputs. Cy3 fluorescence enhancement can be explained by recalling the concept of plasmon-enhanced fluorescence, which is a condition that is fulfilled when a fluorophore emission frequency matches a metal nanoparticle plasmon resonance. As a result, an amplified emission at the same frequency as that of the organic fluorophore is attained. For this purpose, the absorption/emission profile of the fluorophore must significantly overlap with one of the optical features of the metal nanoparticle [32,33]. Moreover, the phenomenon strongly depends on the particle shape, size, and fluorophore-particle separation distance, as observed herein. Conversely, quenching can be ascribed to the instant energy transfer from the excited state of the fluorophore to the peripheral conduction electrons of the metal nanoparticle. The quenching is dependent on the donor-acceptor distance and occurs when a fluorophore is positioned at a distance below 10 nm from the plasmonic surface as in the present case [32].

**Figure 4:**
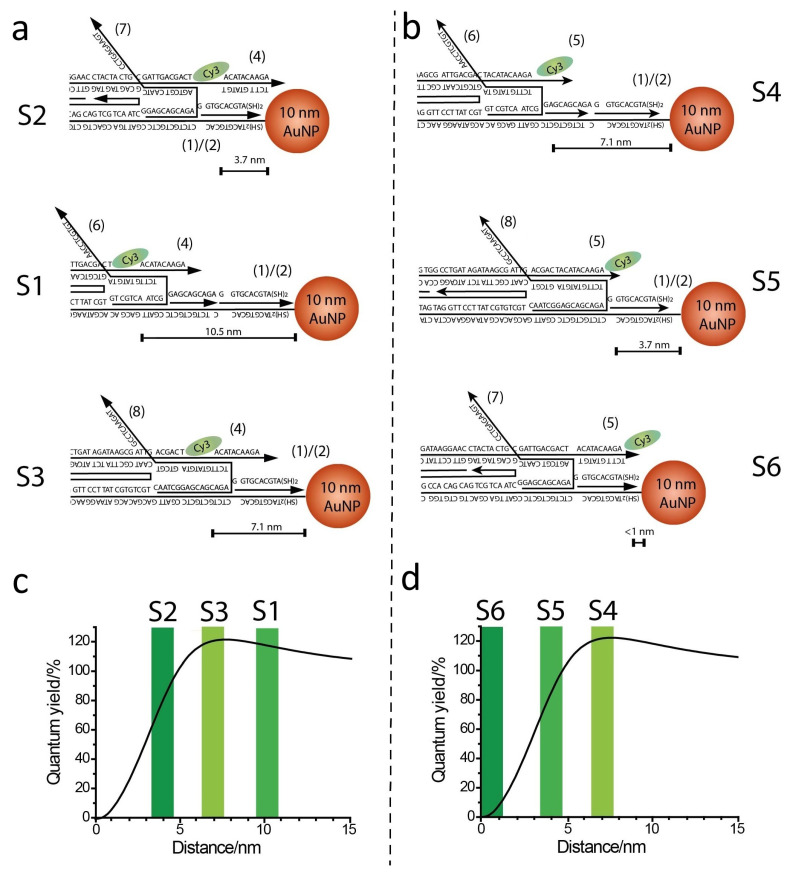
Comparison of experimental and theoretical quenching/enhancement effects. (**a**,**b**) Schemes of geometric distances separating Cy3 and 10 nm AuNP on the slider, as calculated considering DNA scaffold sizes. (**c**,**d**) Theoretical models of the distance-dependent quenching/enhancing effects of a spherical 10 nm-diameter gold plasmonic surface on Cy3 fluorescence emission according to [34].

To address the different emission levels, we used geometrical considerations to calculate the expected Cy3-AuNP distances for the different states. Figure 4a,b shows slider geometric schemes associated with different values for the separating distances that are equal to 10.5, 7.1, and 3.7 nm, associated with states S1, S3, and S2, and distance values equal to 7.1, 3.7, and <1 nm, associated with states S4, S5, and S6 (see also Supplementary Materials for additional details on the calculated distances). These values were compared to the theoretical enhancing/quenching effects using a published model [34] (Figure 4c,d). Indeed, the results are in good agreement with the model, confirming the correct assumptions for the geometric considerations.

## 4. Materials and Methods

A detailed description of the steps required to assemble the nano-ruler is presented in the Supplementary Materials. Briefly, thiolated synthetic DNA oligonucleotides (Integrated DNA Technologies, Coralville, IA, USA) were reacted with AuNPs to obtain the conjugate AuNP-DNA. Electrophoretically separated (LonzaGroup AG, Basel, Switzerland, small-nucleotide separating 2% agarose; separation carried out in ice at low voltage) single DNA carrying AuNPs were then used to self-assemble the nano-ruler by temperature ramp, comprising a Cy3-modified oligonucleotide. The final complex was operated at a concentration of 50 nM with a small excess of reconfiguring strands.

Theoretical calculation correlating the distance-dependent fluorescence quantum yields of the Cy3 fluorophore in the AuNP-rotaxane dumbbell structure. The fluorescence features of Cy3 in the different configurations of the AuNP (10 nm)/DNA/Cy3 hybrid rotaxane system were simulated theoretically by using an on-line, free available software [34]. We attempted to theoretically calculate the fluorescence quantum yields of Cy3 as a function of the distance separating the fluorophore from the NP surface. In order to do that, the following parameters were adopted in the software-assisted calculations:—equal diameter: 10 nm S8—prolate aspect ratio: 1—metal: Au—medium index: 1.3—X-axis distance: 0–30 nm—wavelength: 560—dipole orientation: perpendicular—reference QE: 0.01—method: Gersten—radiative damping: yes—dynamic depolarization: yes—angular mode numbers lstart: 1; leval: 0; and lcut: 60. The final plots are shown in Figure 4c,d, where a maximum enhancement (20%) is predicted at a separating distance of ca. 7 nm and the quenching area is positioned in the separating distance interval of 0–5 nm.

## 5. Conclusions

Fluorescent DNA nano-rulers are dynamic supramolecular systems with the ability to configure a defined number of fluorophores in programmed nano-sized architectures. Herein, the optimization of a switchable DNA nano-machine able to perform cyclic and reversible transitions between discrete states was described. The supramolecular self-assembly consists of a quasi-circular ring acting as a support for two sliding ssDNA rails, with the programmed motion varying the distance between a AuNP and a Cy3 fluorophore. In order to obtain an actual fine-tunable fluorescence emitter, 5, 10, and 15 nm nanoparticles were screened, identifying 10 nm as the optimal AuNP size displaying the best dynamic range, both in terms of quenching and enhancement. The movements are generated by strand displacement reactions due to complementary sequences that are present in the milieu, hence the system can be used as a fluorescent reporter for single-strand nucleic acids such as RNA viruses.

## Data Availability

Data is contained within the article and Supplementary Materials.

## Supplementary Materials

The supporting information can be downloaded at: https://www.mdpi.com/article/10.3390/ijms26062557/s1.

## Abbreviations

The following abbreviations are used in this manuscript:

ssDNA	Single-strand DNA
AuNP	Gold nanoparticle
Cy3	Sulfo-Cyanine3
