# Supplementary material for: A DNA-Based Plasmonic Nano-Ruler
Source: Int J Mol Sci. 2025 Mar 12;26(6):2557. doi: 10.3390/ijms26062557 (PMC11942521; doi:10.3390/ijms26062557)
Supplement: Supplementary file 1 [file ijms-26-02557-s001.zip › ijms-3481759-supplementary.pdf]

Supplementary Information

A DNA-based Dynamic Nano-ruler

Aura Cencini, Mary Bortoluzzi, Graziano Rilievo, Federica Tonolo, Fabio Vianello, Massimiliano Magro, and Alessandro Cecconello*

Department of Comparative Biomedicine and Food Science, viale dell'Università 16, 35020 Legnaro (PD), University of Padova, Italy

Email: massimiliano.magro@unipd.it

1. Materials

Sodium chloride, magnesium chloride, dithiothreitol, acrylamide/bis-acrylamide (30%), bis(p-sulfonatophenyl)phenylphosphine dihydrate dipotassium salt, ethylenediaminetetraacetic acid (EDTA), ammonium persulfate, N,N,N′,N′-Tetramethylethylenediamine (TEMED), sodium dodecyl sulfate (SDS), and tris(2-carboxyethyl)phosphine hydrochloride were purchased from Sigma–Aldrich (Merck). Urea was purchased from J.T. Baker Chemical Co. 10 nm gold colloid was purchased from BBI Solutions. "Metaphor" agarose was purchased from Lonza. 10 kDa MWCO "Snakeskin" dialysis tubings were purchased from Thermo Scientific. Amicon ultra 100 kDa MWCO were purchased from Millipore. 0.22 µm disposable filter units were purchased from Whateman GmbH. Amersham G-25 microspin columns were purchased from GE Healthcare. All DNA oligonucleotides were purchased from Integrated DNA Technologies, IDT, (Table S1). Ultrapure water from a NANOpure Diamond (Barnstead) source was used in all experiments.

Table S1. Sequences of the core structure oligonucleotides

(1)	$(SH)_2$-TACGTGCACCTCTGCTGCTCCGATTGACGACACGATAAGGAACCTACTACT GCGATTGACGAC TACATACAAGA
(2)	GTGCACGTA-$(SH)_2$
(3)	AGGCCACAGCAGTCGTCAATCGCAGTAGTAGGTTCCTTATCGTGTCGTCAATCGCTTATCTATC
(4)	CTCTGCTGCTCCGATTGACGACTGCTGTGGCCTGATAGATAAGCGATTGACGAC-Cy3-ACATACAAGA
(5)	CTCTGCTGCTCCGATTGACGACTGCTGTGGCCTGATAGATAAGCGATTGACGAC TACATACAAGA-Cy3

Table S2. Sequences of the operating and rigidifying oligonucleotides. Star-signs indicate complementary sequences.

(6)	GTCGTCAATCGTCTTGTATGTAAACCTCGTGT
(6*)	ACA CGA GGT TTA CAT ACA AGA CGA TTG ACG AC
(7)	GGAGCAGCAGAAGTCGTCAATCCCTGAGAAGT
(7*)	ACT TCT CAG GGA TTG ACG ACT TCT GCT GCT CC
(8)	CAATCGGAGCAGCACATCTTGTATGTAGTCGTGCCTCAAGAT

(8*)	ATC TTG AGG CAC GAC TAC ATA CAA GAT GTG CTG CTC CGA TTG
(9)	GAGCAGCAGA
(10)	TCTTGTATGT
(11)	TTTTT-SH

2. Pre-functionalization of commercial AuNPs and preparation of dithiol DNA stock solution

10 nm AuNPs, as provided by the manufacturer, were mixed with an excess of Bis(p-sulfonatophenyl)phenylphosphine dihydrate dipotassium salt (BSPP) and stirred for 8 hours. The resulting Au NPs were, then, concentrated using Amicon filtering tubes 100 kDa MWCO, for 5 min under 3000g to a final concentration of 1-10 µM (if necessary, a second concentration step was performed using Amicon filtering tubes). AuNP concentration was estimated by diluting the resulting solution (1:1000), recording the absorption spectrum, and using AuNP extinction coefficient at 520 nm. The resulting AuNP solution was stored at +4°C, and used for several days. Dithiol-functionalized DNA strands (1) and (2), were diluted to a final concentrations of 15 µM with 15 mM Tris(2-carboxyethyl)phosphine hydrochloride (TCEP). DNA solutions were stored at -20°C and used for several weeks. Prior to each use, AuNPs were subjected to desalting to remove excess TCEP using Amersham microspin G-25 columns, following manufacturer instructions.

3. Conjugation of AuNPs and thiolated nucleic acid strands

AuNP-functionalized Rail A was assembled as follows: Thiolated strands (1) and (2), as prepared in point 3, were mixed in a solution that included 0.5X TBE, 0.4 mg/mL BSPP, and 100 mM NaCl. The resulting mixture was incubated at 90 °C for 5 min, and cooled down slowly to 4 °C and, resulting in the hybridization of (1) and (2). Subsequently, 10 nm AuNPs, as prepared in point 3, was added to the solution. The mixture was, then, incubated for 2 hours at room temperature lo allow conjugation of the thiol to the gold surface and then separated by gel electrophoresis (see next paragraph, point 4).

4. Purification of the AuNP-functionalized Rail A.

Single duplex (1)/(2)-functionalized AuNPs were separated from the polyconjugated DNA-AuNPs mixture, and the unreacted AuNPs, on a 3% w/v agarose gel (MetaPhor). Samples were mixed with 12 % v/v glycerol

(final concentration), and loaded into the wells of the gel. The gels were run at a constant voltage of 80 V. When satisfactory separation was observed, the bands of interest were excised (see Figure 1A in the main text) and subjected to a voltage of 100 V inside a closed dialysis membrane filled with 0.5X TBE buffer (in order to release the desired modified NPs from the gel). Then, the solution trapped in the dialysis membrane was collected, filtered with a 0.22 µm syringe filter (Whatman), and concentrated with a 100 kDa MWCO Amicon filtering tube.

5. Stabilization of the AuNP-functionalized (1)/(2) duplex.

The separated AuNP-functionalize duplex displayed limited stability, and precipitated with time. To eliminate this difficulty, the AuNP-functionalize duplex was stabilized with an excess of a short thiolated DNA oligo (11) in a solution that included 100 mM NaCl, 0.5X TBE and 0.4 mg/mL BSPP. The resulting mixture was incubated at 25°C for 48 h and then washed four times using 100 kDa MWCO Amicon filtering tubes 5 min × 3000g, while diluting the samples to 15 mL in water each time, in order to remove (11) excess. At the end, the stabilized AuNP-functionalized (1)/(2) was diluted (1:120) and its concentration was determined by recording the absorption spectrum and using the appropriate AuNP extinction coefficient at 520 nm.

6. Assembly and operation of the AuNP-functionalized nano-ruler.

An excess of stabilized DNA-conjugated AuNPs were mixed with strands (4), or (5), and (3) at a final concentration of 1 µM in a ratio 1:1:1 in 500 mM NaCl, TBE 0.5X. The mixture was heated at 70°C for 5 minutes in a thermal cycler and cooled down slowly to 4°C in 4 hours. Reconfigurations were operated directly in a quartz cuvette (Hellma GmbH, QS, volume= 120 µL) at room temperature, and at a slider concentration of 100 nM, while recording fluorescence emission at 565 nm (excitation wavelength equals 555 nm). Initially, the emission signal was recorded continuously until it was stable. At this point, stepwise additions of the reconfiguration strands were carried out with increasing volumes starting at 0.5 µL to obtain final dilutions of the operating strands equal to the slider concentration X1.2 and limited changes in the overall volume (e.g., first, second, and third additions reached final concentrations equal to 120, 144, and 173 nM, respectively). Final emission levels reported in Figures 2 and 3, in the main text, represent average values calculated over 2.5 minutes with error bars representing standard deviations.

8. Theoretical calculations correlating the distance-dependent fluorescence quantum yields of the Cy3 fluorophore in the AuNP-functionalized nano-ruler structure.

The fluorescence features of Cy3 in the six configurations of the AuNPs(10nm)/DNA/Cy3 hybrid slider system were simulated theoretically by using an on-line, free available software (H. Mertens, A. F. Koenderink, A. Polman, Phys. Rev. B 2007, 76, 115123-115134. http://erbium.amolf.nl/). We attempted to theoretically calculate the fluorescence quantum yields of Cy3 as a function of the distance separating the fluorophore from the NP surface. In order to do that, the following parameters were adopted in the software-assisted calculations:

- equal diameter: 10 nm
- prolate aspect ratio: 1
-metal: Au
-medium index: 1.3
- X-axis distance: 0-30 nm
- wavelength: 565 nm
- dipole orientation: perpendicular
-reference QE: 0.01 -method: Gersten
- radiative damping: yes
- dynamic depolarization: yes
- angular mode numbers lstart: 1; leval: 0 and lcut: 60

The final plots are shown in Figure 4, in the paper, where a maximum enhancement (20%) is predicted at a separating distance of ca. 7 nm and the quenching area is positioned in the separating distance interval of 0-5 nm. Geometric distances (Pythagorean diagonals) were calculated as derived from the DNA scaffolds, considering a rigid DNA duplex with a base-pair length of 0.34 nm along the duplex axis and a duplex thickness of 2.4 nm.

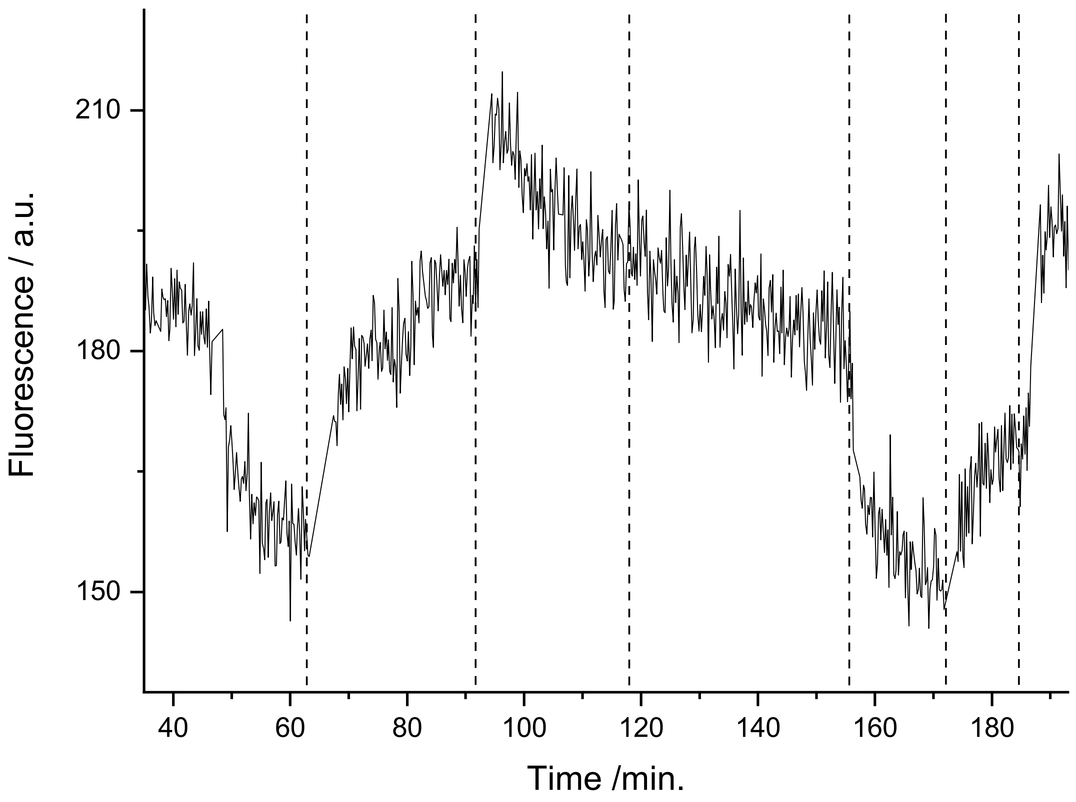

Figure s1. Mechanical control through strand displacement reactions of the fluorescence properties of the DNA slider comprising 15nm AuNP Rail A and ssDNA (4) Rail B. Fluorescence emission levels recorded at (λ= 565 nm) associated with the slider reconfigured across states S1, S2, S1, S3, S1, S2, S1, S3.
